# Supplementary material for: DNA-Functionalized Nanoparticles for Multicolor Cathodoluminescence Imaging
Source: bioRxiv. 2026 Apr 9:2026.04.07.716901. Preprint. [Version 1] doi: 10.64898/2026.04.07.716901 (PMC13082009; doi:10.64898/2026.04.07.716901)
Supplement: Supplement 1 [file media-1.pdf]

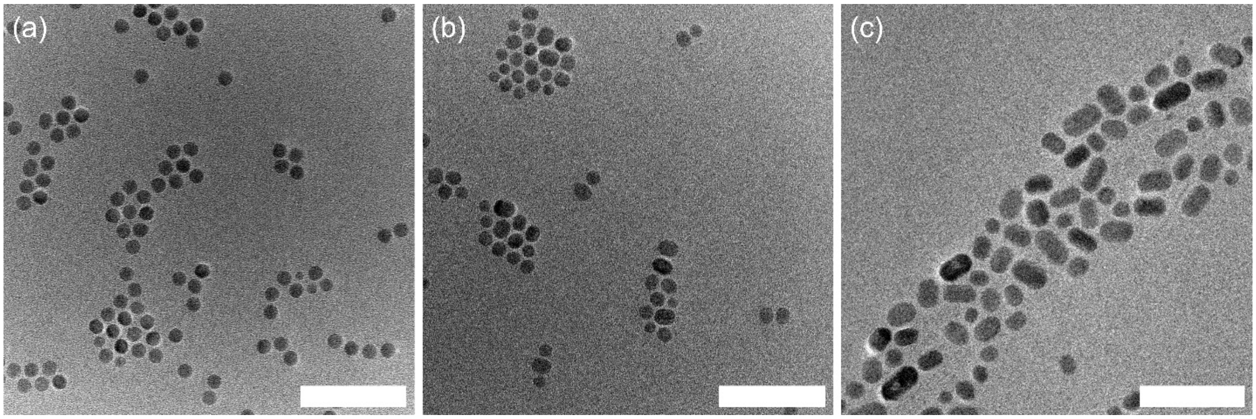

Supplementary Figure 1: TEM images of LNPs. TEM characterization of **(a)** $NaHo_{0.8}Lu_{0.2}F_4$, **(b)** $NaDy_{0.8}Lu_{0.2}F_4$, and **(c)** $NaTbF_4$ LNPs. Images were acquired at 200 keV beam energy using a JEOL JEM-F200 S/TEM in TEM mode. Scale bars: 100 nm.

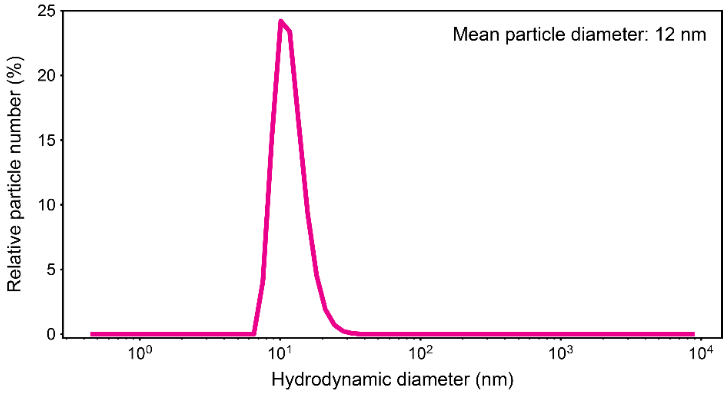

Supplementary Figure 2: DLS with hexane-suspended LNPs. Particle number distribution from DLS measurements of $NaHo_{0.8}Lu_{0.2}F_4$ LNPs in n-hexane. Mean particle diameter was 12.0 ± 3.2 nm.

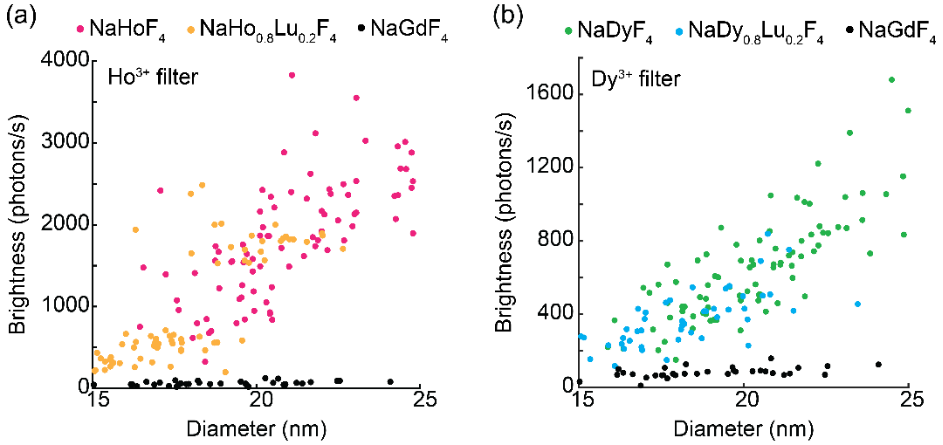

Supplementary Figure 3: Brightness of LNPs when co-doped with Lu^{3+} ions. (a) Brightness of $NaHoF_4$ and $NaHo_{0.8}Lu_{0.2}F_4$ LNPs in the Ho^{3+} filter as a function of LNP diameter. **(b)** Brightness of $NaDyF_4$ and $NaDy_{0.8}Lu_{0.2}F_4$ LNPs in the Dy^{3+} filter as a function of LNP diameter. Brightness of control $NaGdF_4$ LNPs is also shown in the respective filters for comparison.
